# Knockout of signal peptide peptidase in the eye reduces HSV-1 replication and eye disease in ocularly infected mice

**DOI:** 10.1371/journal.ppat.1010898

**Published:** 2022-10-10

**Authors:** Shaohui Wang, Ujjaldeep Jaggi, Homayon Ghiasi

**Affiliations:** Center for Neurobiology & Vaccine Development, Ophthalmology Research, Department of Surgery, Cedars-Sinai Medical Center, Los Angeles, California, United States of America; University of Illinois at Chicago, UNITED STATES

## Abstract

We previously reported that knocking out signal peptide peptidase (SPP), a glycoprotein K (gK) binding partner, in mouse peripheral sensory neurons reduced latency-reactivation in infected mice without affecting primary virus replication or eye disease. Since virus replication in the eye plays an essential role in eye disease, we generated a conditional knockout mouse lacking SPP expression in the eye by crossing Pax6 (paired box 6)-Cre mice that have intact Pax6 expression with SPP^flox/flox^ mice. Significantly less SPP protein expression was detected in the eyes of Pax6-SPP^-/-^ mice than in WT control mice. HSV-1 replication in the eyes of Pax6-SPP^-/-^ mice was significantly lower than in WT control mice. Levels of gB, gK, and ICP0 transcripts in corneas, but not trigeminal ganglia (TG), of Pax6-SPP^-/-^ infected mice were also significantly lower than in WT mice. Corneal scarring and angiogenesis were significantly lower in Pax6-SPP^-/-^ mice than in WT control mice, while corneal sensitivity was significantly higher in Pax6-SPP^-/-^ mice compared with WT control mice. During acute viral infection, absence of SPP in the eye did not affect CD4 expression but did affect CD8α and IFNγ expression in the eye. However, in the absence of SPP, latency-reactivation was similar in Pax6-SPP^-/-^ and WT control groups. Overall, our results showed that deleting SPP expression in the eyes reduced primary virus replication in the eyes, reduced CD8α and IFNγ mRNA expression, reduced eye disease and reduced angiogenesis but did not alter corneal sensitivity or latency reactivation to HSV-1 infection. Thus, blocking gK binding to SPP in the eye may have therapeutic potential by reducing both virus replication in the eye and eye disease associated with virus replication.

## Author summary

Interaction of HSV-1 gK and signal peptide peptidase (SPP) plays an essential role in HSV-1 infectivity. To evaluate the importance of gK binding to SPP in the eye following primary virus replication and latency-reactivation, we evaluated SPP in the eye using Pax6-SPP^-/-^ conditional knockout mice. The absence of SPP in the eye significantly reduced primary virus replication and eye disease but does not affect latency-reactivation.

## Introduction

Herpes simplex virus type-1 (HSV-1) is the leading cause of infectious blindness in developed countries, with an estimated 8.4 to 13.2 new cases per 100,000 people/year [1]. The rates of ocular HSV reactivation increase with increasing years of ocular infection [2]. Virus replication in the eyes of infected individuals is of considerable clinical importance especially with respect to eye disease. Due to the severe consequences of ocular infection, reducing virus replication in the eyes is a major goal in the effort to control ocular HSV infection and subsequent eye disease. Development of effective strategies to limit virus replication in the eye has been limited by an insufficient understanding of mechanisms that regulate intermittent reversion from the latent to the infectious state, including regulation of the immune response to HSV infection, which is known to be a major contributing factor to eye disease [3–5]. Thus, there is a critical need to develop alternative approaches to prevent and control serious HSV-induced ocular diseases. Although it is well established that immune responses to HSV-induce corneal scarring (CS), the exact identity of the immune responses that lead to CS has not been precisely determined despite extensive research in this area. To address this important issue, we have focused on identifying molecular features of the virus that drive the disease process. Of the more than 80 HSV genes, at least 12 encode glycoproteins and some of these glycoproteins are major inducers and targets of humoral- and cell-mediated immune responses following herpes infection [6–10]. Amongst these 12 known glycoproteins, we have previously demonstrated that HSV glycoprotein K (gK), and no other HSV glycoproteins, is involved in exacerbating eye disease and that this enhancement of eye disease is independent of mouse or virus strain [8,10–17].

Thus, our published studies strongly suggest that gK is the viral protein that exacerbates eye disease in mice [8,14,18] and humans [11], supporting the concept that gK plays a major role in viral immunopathogenesis. gK is known to bind to signal peptide peptidase (SPP) and this binding has been shown to be essential for HSV infectivity *in vitro* using SPP dominant-negative mutants and shRNA against SPP [19]. The binding of gK to SPP can be blocked *in vitro* by SPP inhibitors such as L685,458, (Z-LL)_2_ ketone, aspirin, ibuprofen, or DAPT [20]. Further, administration of (Z-LL)_2_ ketone as an eye drop reduced primary HSV infectivity and eye disease in ocularly infected mice [20]. Similar to gK [21,22], SPP is also an essential gene and mice lacking SPP are embryonically lethal [23,24]. We previously generated an SPP-inducible knockout mouse strain using a tamoxifen-inducible Cre recombinase and showed that the absence of SPP reduced primary HSV replication in the eyes and latency in ocularly infected mice [24]. Thus, despite the incomplete depletion of SPP by tamoxifen in the knockout mice, depletion of SPP significantly reduced both primary and latent infection in depleted mice. Since peripheral sensory neurons are the sites of HSV latency and reactivation [25–27], we recently constructed conditional knockout mice in which SPP was specifically deleted in mouse peripheral sensory neurons [28]. As expected, the absence of SPP in peripheral sensory neurons of infected mice did not affect primary virus replication in the eyes but significantly reduced latency-reactivation in trigeminal ganglia (TG) of infected mice [28].

The eye is the site of primary HSV infection and following reactivation, virus travels back from TG to the eye to cause eye disease. Since, SPP deletion in peripheral sensory neurons is known to affect virus infection and eye disease, we asked if SPP deletion in the eye would have a similar effect. Using the eye-specific *Pax6* (paired box 6) gene, which is essential for eye development and is expressed in all eye tissues [29], we generated a Cre-driver mouse line that expresses Cre recombinase from the Pax6 gene locus [30,31]. In the present study, we used this conditional knockout mouse strain *(*Pax6-SPP^-/-^), which *lacks SPP expression in the eye* and to determine whether SPP expression in the eye is required for HSV infectivity. We found that in the absence of SPP expression in the eye: 1) virus replication was significantly reduced in the eyes of infected Pax6-SPP^-/-^ mice than in WT control mice; 2) latency and reactivation in infected Pax6-SPP^-/-^ mice was similar to that in WT control mice; and 3) corneal scarring and angiogenesis in infected Pax6-SPP^-/-^ mice at various time points post infection was significantly lower than in WT control mice, and corneal sensitivity was significantly higher in the absence of SPP than in WT control mice. Consequently, the absence of SPP in the eyes of infected mice was associated with reduced virus replication and less eye disease. Based on our current and previous studies [19,20,24,28], we have now identified tissue specific interactions of gK with SPP that appears to play critical roles in viral replication, eye disease, and latency-reactivation.

## Results

### Generation of Pax6-SPP^-/-^ mice

**Fig 1:**
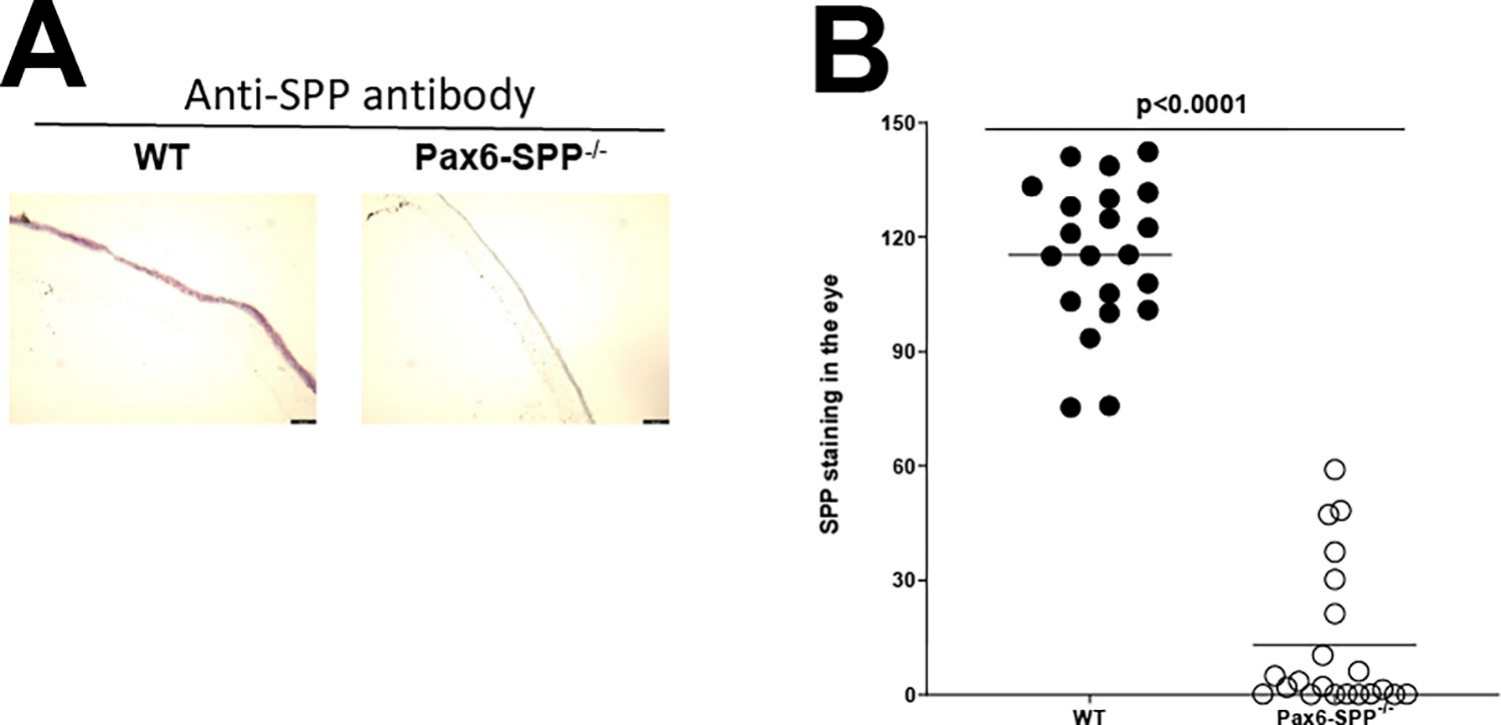
Development of Pax6 promoter-driven Cre specific SPP conditional knockout mice. A) Confirmation of lack of SPP expression in the eyes of Pax6-SPP^-/-^ mice by IHC. Pax6-SPP^-/-^ mice were generated by crossing SPP^flox/flox^ mice with Pax6-Cre mice to generate Pax6-SPP^-/-^ mice, in which SPP was knocked out in Pax6 expressing tissue as described in Materials and Methods. Eyes from two-month-old Pax6-SPP^-/-^ mice or WT control mice were harvested and frozen in OCT at -80°C until further processing. IHC was performed using an anti-SPP antibody and developed using a VectaShield VIP substrate kit. SPP positive cells show purple color. SPP expression was notably reduced in the eye of Pax6-SPP^-/-^ mice (right panel) but was readily detected in the eye of WT control mice (left panel), specifically on the retinal side. As shown, less SPP staining was observed in the Pax6-SPP^-/-^ group than in the WT group. B) Quantification of SPP staining density. SPP stained sections of WT (closed circles) and Pax6-SPP^-/-^ (open circles) mouse eyes were quantified using ImageJ software. A total of 21 photos from six eyes per group are plotted. Size bar 50um.

Using combinations of SPP dominant-negative mutants, SPP shRNA, chemical inhibitors of SPP, tamoxifen depletion of SPP, and mice lacking SPP in their peripheral sensory neurons, we have shown the important role of HSV gK binding to SPP in HSV infectivity *in vitro* and *in vivo* [19,20,24,28]. In our model of ocular HSV infection, eyes are the first site of virus infection, thus we wanted to determine how the absence of SPP affected HSV infectivity in the eyes *in vivo*. To address this question, we generated mice lacking SPP in their eyes by crossing SPP^flox/flox^ mice with mice expressing Cre recombinase under control of the Pax6 promoter [30,31]. Breeding of these mice produced mice lacking SPP expression in the eye, referred to as Pax6-SPP^-/-^ mice (see Materials and Methods). SPP protein expression was monitored by staining eye sections from Pax6-SPP^-/-^ and WT control mice with anti-SPP antibody (Fig 1A). We observed less positive SPP staining in eye sections from Pax6-SPP^-/-^ mice than in eye sections from WT control mice (Fig 1A). As reported previously [30,31], SPP was expressed more strongly in retina than cornea of WT mice (Fig 1A, eye section from WT control group). Similar to this study, previously it was reported that retina expresses more SPP than many other parts of mouse brain (https://www.proteinatlas.org/ENSG00000101294-HM13/summary/rna).

Density of SPP expression in these IHC stained eye sections was quantified as described in Materials and Methods. Significantly less SPP expression was observed in eye sections from Pax6-SPP^-/-^ mice than in eye sections from WT control mice (Fig 1B, p<0.001). Thus, Pax6-SPP^-/-^ mice have notably less SPP expression in their eyes. These mice grow normally, and no eye dysfunction was detected in these mice using esthesiometry and tonometry.

### Virus replication in the eyes of ocularly infected Pax6-SPP^-/-^ mice was reduced by the absence of SPP

**Fig 2:**
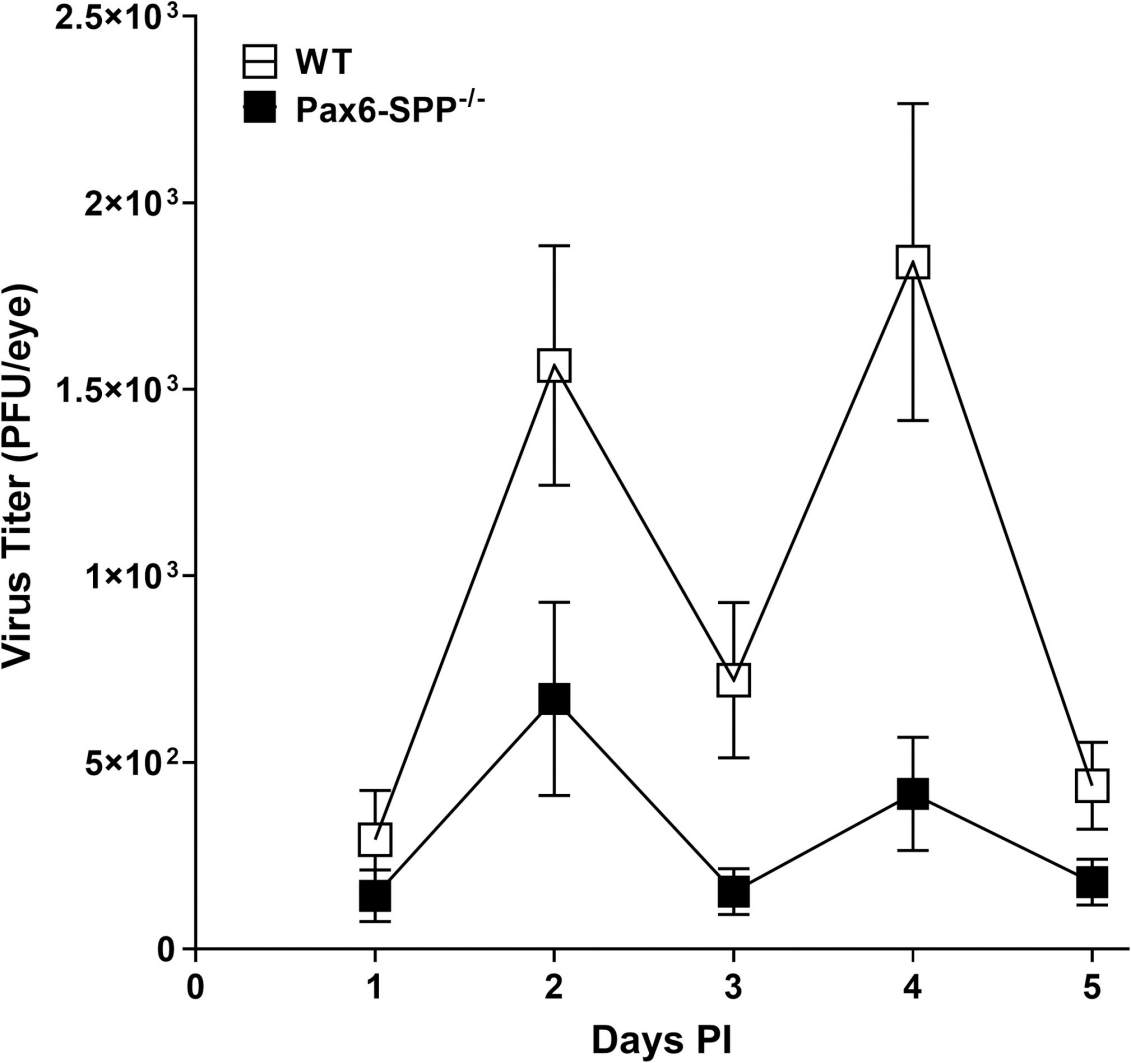
Virus titer in the tear of infected Pax6-SPP^-/-^ mice. Pax6-SPP^-/-^ and WT control mice were ocularly infected with 2 X 10^5^ pfu/eye of HSV strain McKrae. Tear films were collected on days 1 to 5 PI and virus titers were determined by standard plaque assays. Each point represents the mean titer of 20 eyes.

To determine if the absence of SPP expression in the eye alters virus replication in the eyes of infected mice, Pax6-SPP^-/-^ and WT control mice were ocularly infected with HSV strain McKrae. Tear films were collected from 20 eyes/group in two separate experiments on days 1, 2, 3, 4, and 5 PI and the presence of infectious virus was determined by plaque assay (Fig 2). Virus titers in the eyes of Pax6-SPP^-/-^ mice on day 1 PI was similar to WT control mice (Fig 2, p>0.05), while virus titers in the eyes of Pax6-SPP^-/-^ mice on days 2–4 was significantly lower than in WT control mice (Fig 2, p<0.05). Thus, the absence of SPP in Pax6-SPP^-/-^ mice was associated with reduced virus replication in the eyes of infected mice. This result is consistent with our previous work showing that the global absence of SPP in tamoxifen-inducible knockout mice was associated with reduced virus replication in the eyes of infected mice [24]. Furthermore, we previously reported that inhibitors of SPP reduced virus replication in the eyes of ocularly infected mice [20].

### Expression of gB, gK, and ICP0, transcripts are reduced in the cornea but not TG of Pax6-SPP^-/-^ mice during primary ocular infection

**Fig 3:**
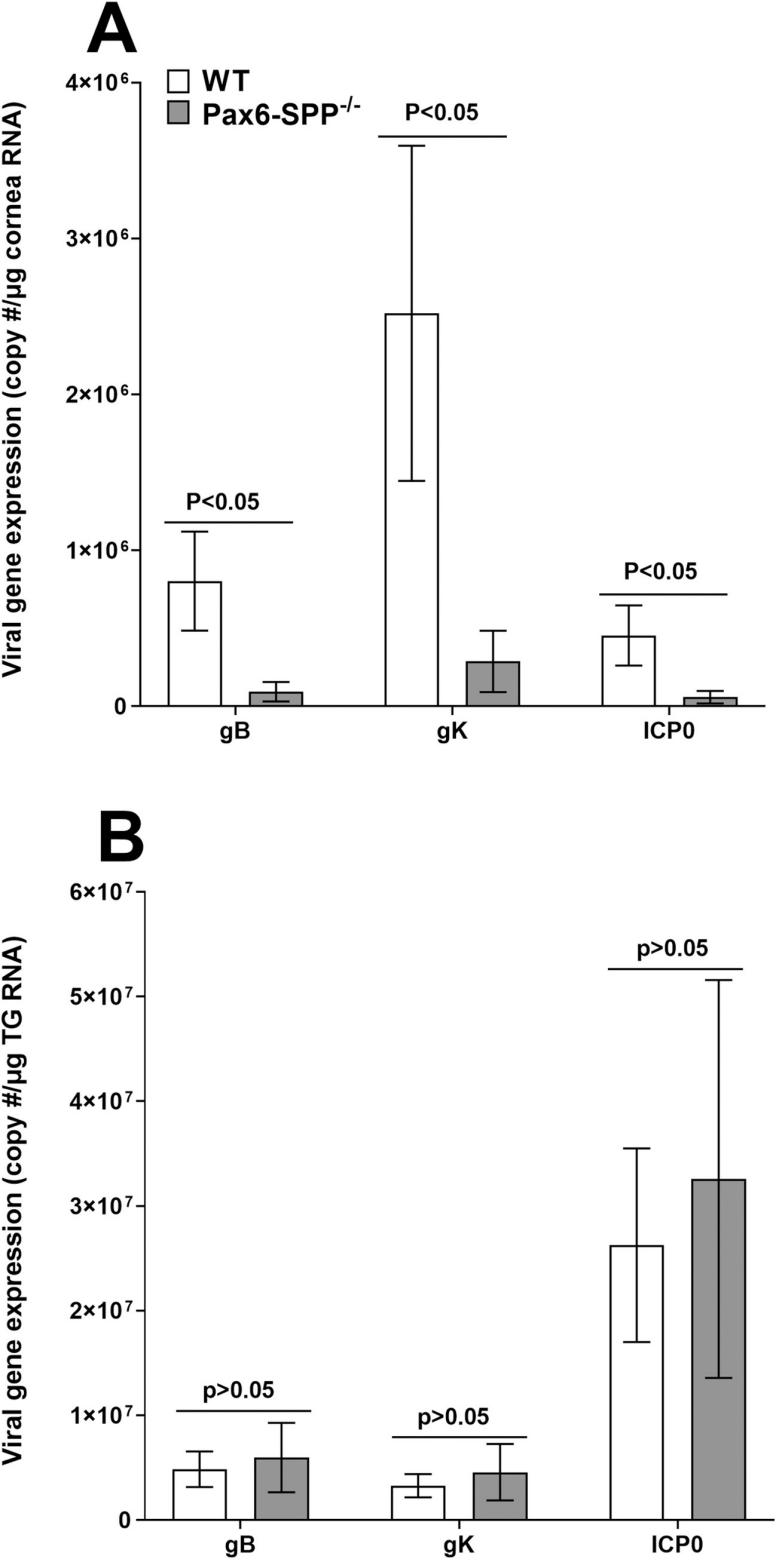
Expression of gB, gK, and ICP0 transcripts in corneas and TG of infected mice. Pax6-SPP^-/-^ (gray bars) and WT control mice (open bars) were ocularly infected with 2 X 10^5^ pfu/eye of HSV strain McKrae. Corneas and TG were harvested on day 4 PI. RNA from corneas (panel A) or TG (panel B) were isolated as described in Materials and Methods. gB, gK, and ICP0 transcripts were measured by qRT-PCR and copy numbers for each transcript were calculated using a standard curve generated from their respective plasmid. Each bar represents the mean ± SEM from 10 corneas or 10 TG.

As expected, Fig 2 showed that the absence of SPP in the eye reduced virus replication in the eyes of infected mice. To confirm our virus replication data in the eyes of infected mice and determine the effect of absence of SPP on gK expression, we measured expression of gK as well as gB and ICP0 transcripts in corneas of infected mice. Pax6-SPP^-/-^ and WT control mice were infected as above with HSV McKrae. The peak difference in virus replication in the eyes of Pax6-SPP^-/-^ and WT control mice was on day 4 PI (Fig 2A). Thus, on day 4 PI, corneas and TG were collected from infected mice and their RNA was isolated. RNA isolated from corneas and TG on 4 days PI was analyzed by TaqMan RT-PCR to determine gK, gB, and ICP0 mRNA copy number (Fig 2A). GAPDH mRNA in each sample was used as an internal control. The results showed significantly lower levels of each transcript in corneas of infected Pax6-SPP^-/-^ mice than in WT control mice (Fig 3A, for all three viral transcripts, p<0.05, Fisher’s exact test). However, while levels of gB, gK, and ICP0 transcripts were significantly lower in Pax6-SPP^-/-^ mouse cornea than in WT control mice, these transcripts did not differ significantly in RNA isolated from TG of infected mice (Fig 3B, p>0.05, for all three viral transcripts, Fisher’s exact test). Collectively, these results indicated that the absence of SPP in corneas of infected mice is associated with reduced viral transcripts in corneal cells but not in TG. Pax6 is known to be expressed in various regions of the developing central nervous system (CNS) [32], but our results suggest that in contrast to the effects of SPP absence on virus replication in the eye, the absence of SPP in infected Pax6-SPP^-/-^ mice did not affect viral transcript expression in TG. These results are consistent with our previous results showing that the effect of SPP depletion on HSV infectivity is tissue specific [28].

### Effect of SPP absence on CD4, CD8α, and IFNγ expression in corneas of infected Pax6-SPP^-/-^ mice during primary infection

**Fig 4:**
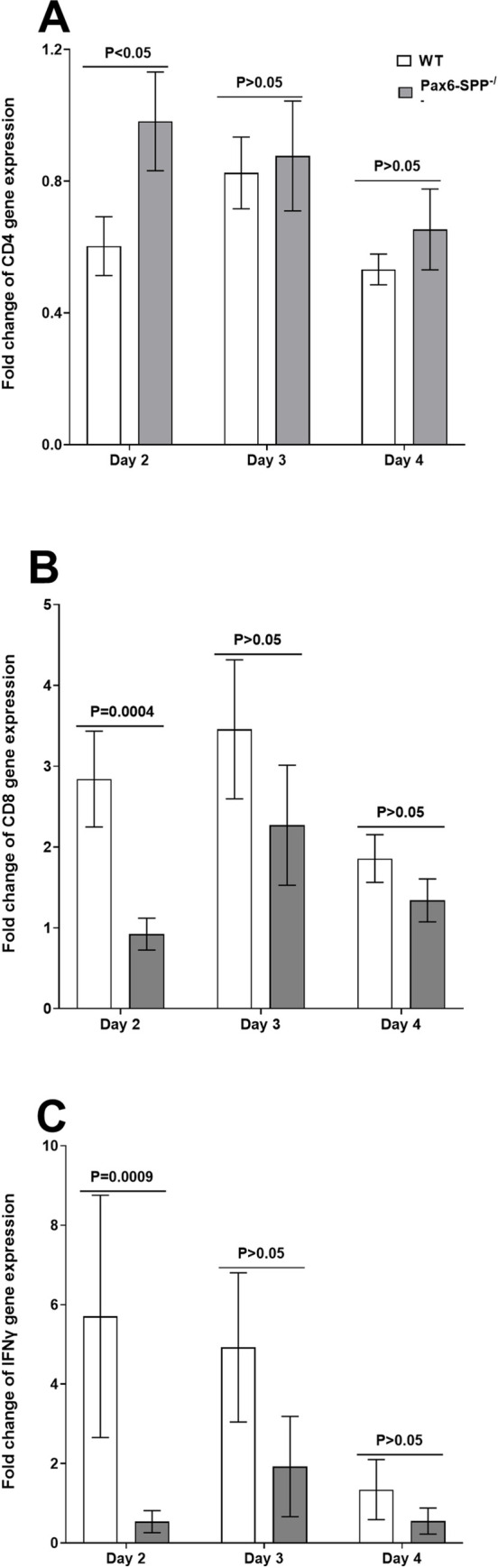
Expression of CD4, CD8α, and IFNγ in the corneas of infected mice. Pax6-SPP^-/-^ (gray bars) and WT mice (open bars) were infected with HSV strain McKrae and corneas were harvested on days 2, 3, and 4 PI. CD4 (panel A), CD8α (panel B), and IFNγ (panel C) expression levels were measured by RT-qPCR. The ratio of each mRNA transcript expression was normalized to its expression in the respective uninfected group. GAPDH expression was used to normalize relative expression of each transcript. Each bar represents the mean ± SEM from 12 corneas from six mice.

The above results suggest that the absence of SPP affects virus replication and viral transcripts in the eyes but not in TG of Pax6-SPP^-/-^ infected mice while the absence of SPP did not significantly affect viral replication or transcripts in WT control mice (Figs 2 and 3). Therefore, we asked whether reduced virus replication and viral transcript levels in the eyes of Pax6-SPP^-/-^ mice affects the expression of CD4, CD8α, and IFNγ transcripts in infected mouse corneas. Pax6-SPP^-/-^ and WT control mice were infected as above with HSV-1 McKrae and RNA was isolated from infected mouse corneas collected on days 2, 3, and 4 PI. RNA isolated from Pax6-SPP^-/-^ and WT control mice was used to measure CD4, CD8α, and IFNγ transcript levels in corneas of infected mice by qRT-PCR. Results are presented as “fold” change over baseline mRNA levels in corneas of naive mice for each group. GAPDH mRNA in each sample was used as an internal control. CD4 transcripts in isolated corneas on day 2 PI were significantly lower in WT control mice than in Pax6-SPP^-/-^ mice (Fig 4A, p<0.05, Fisher’s exact test), while no significant differences in mRNA expression were detected between Pax6-SPP^-/-^ and WT control mice on days 3–4 PI (Fig 4A, p>0.05, Fisher’s exact test). In contrast to CD4 transcripts (Fig 4A), significantly lower levels of CD8α transcripts were observed in Pax6-SPP^-/-^ mouse corneas on day 2 PI than in WT control mice (Fig 4B, p = 0.0004, Fisher’s exact test), while no significant differences in mRNA expression levels were detected between Pax6-SPP^-/-^ and WT control mice on days 3–4 PI (Fig 4B, p>0.05, Fisher’s exact test). Finally, similar to CD8α transcripts (Fig 4B), IFNγ transcript levels in Pax6-SPP^-/-^ mouse corneas were significantly lower on day 2 PI than in WT control mice (Fig 4C, p<0.0009, Fisher’s exact test). However, no significant differences in expression levels were detected between Pax6-SPP^-/-^ and WT control mice on days 3–4 PI (Fig 4C, p>0.05, Fisher’s exact test). These results suggest that absence of SPP in the eye did not affect CD4 expression but did affect CD8α and IFNγ mRNA expression in the eye during acute viral infection. Thus, lower CD8α and IFNγ mRNA expression correlates with reduced virus replication in the eye.

### Absence of SPP in the eye reduces corneal sensitivity to HSV infection

**Fig 5:**
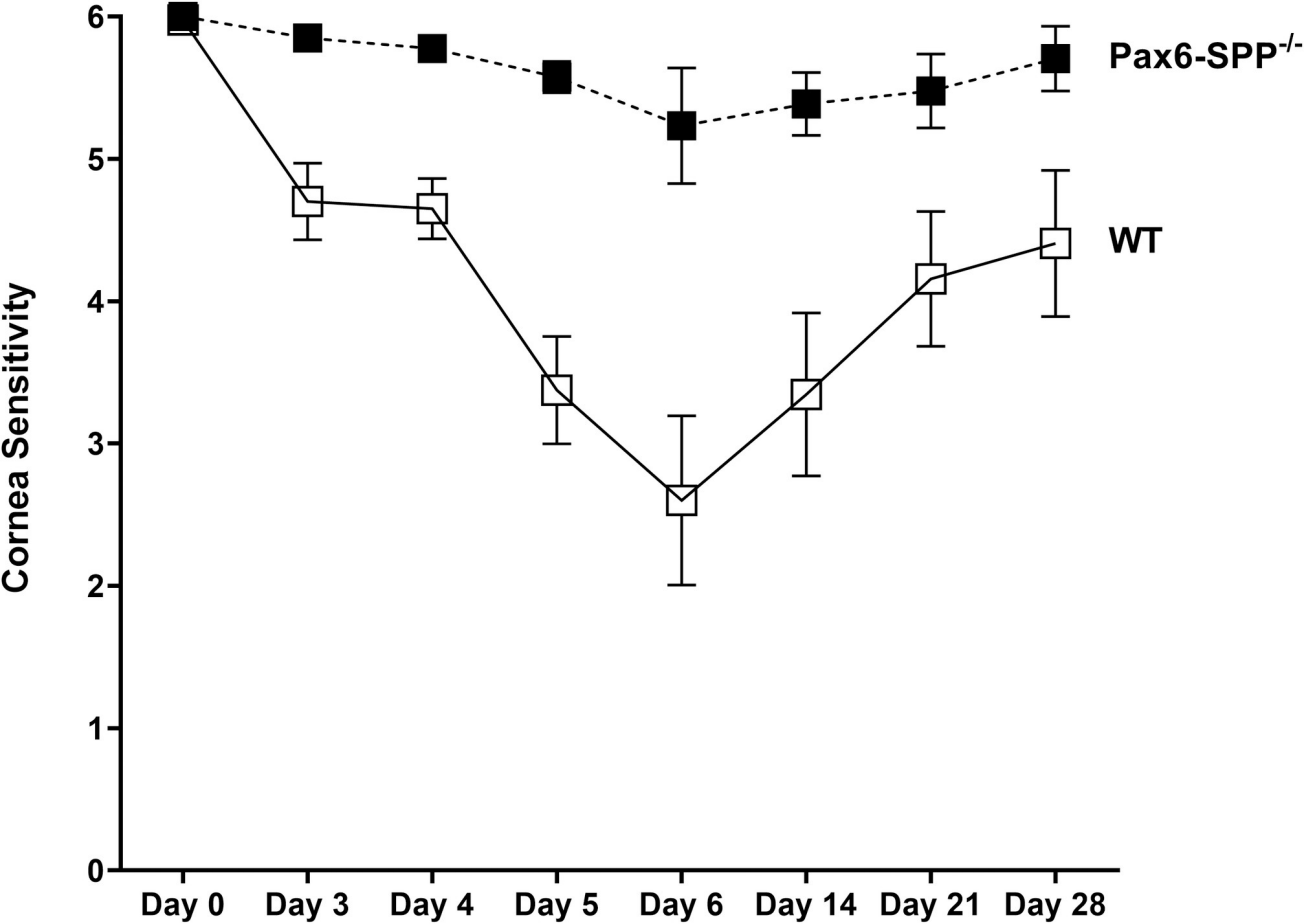
Effect of SPP deficiency on corneal sensitivity in infected mice. Pax6-SPP^-/-^ and WT control mice were ocularly infected with 2 X 10^5^ pfu/eye of HSV strain McKrae. Corneal sensitivity was measured at various times using a Cochet Bonnet aesthesiometer as described in Materials and Methods. Corneal sensitivity was determined using 20 eyes for WT mice (open boxes) and 22 eyes for Pax6-SPP^-/-^ mice (closed boxes).

Ocular HSV infection has been shown to reduce corneal sensitivity [33,34]. To determine how reduced HSV replication in the eyes of Pax6-SPP^-/-^ mice affects corneal sensitivity, a total of 35 Pax6-SPP^-/-^ mice and 35 WT control mice (in three separate experiments) were ocularly infected with 2 X 10^5^ pfu/eye of HSV strain McKrae as above. Corneal sensitivity in infected mice was measured before infection (day 0) and on days 3, 4, 5, 6, 14, 21, and 28 PI as described in Materials and Methods. On all tested days PI, corneal sensitivity did not differ significantly from that on day 0 in Pax6-SPP^-/-^ infected mice (Fig 5). In contrast, corneal sensitivity significantly declined in WT control mice from day 0 to day 6 PI and sensitivity restored slowly after that (Fig 5). However, at all indicated time points PI, corneal sensitivity was lower in WT control mice than in Pax6-SPP^-/-^ mice (Fig 5, p<0.05 on days 3 to 28 PI), suggesting that absence of SPP in the eye correlates with reduced virus infectivity and thus higher corneal sensitivity.

### Absence of SPP in the eye reduces corneal scarring and angiogenesis in infected mice

**Fig 6:**
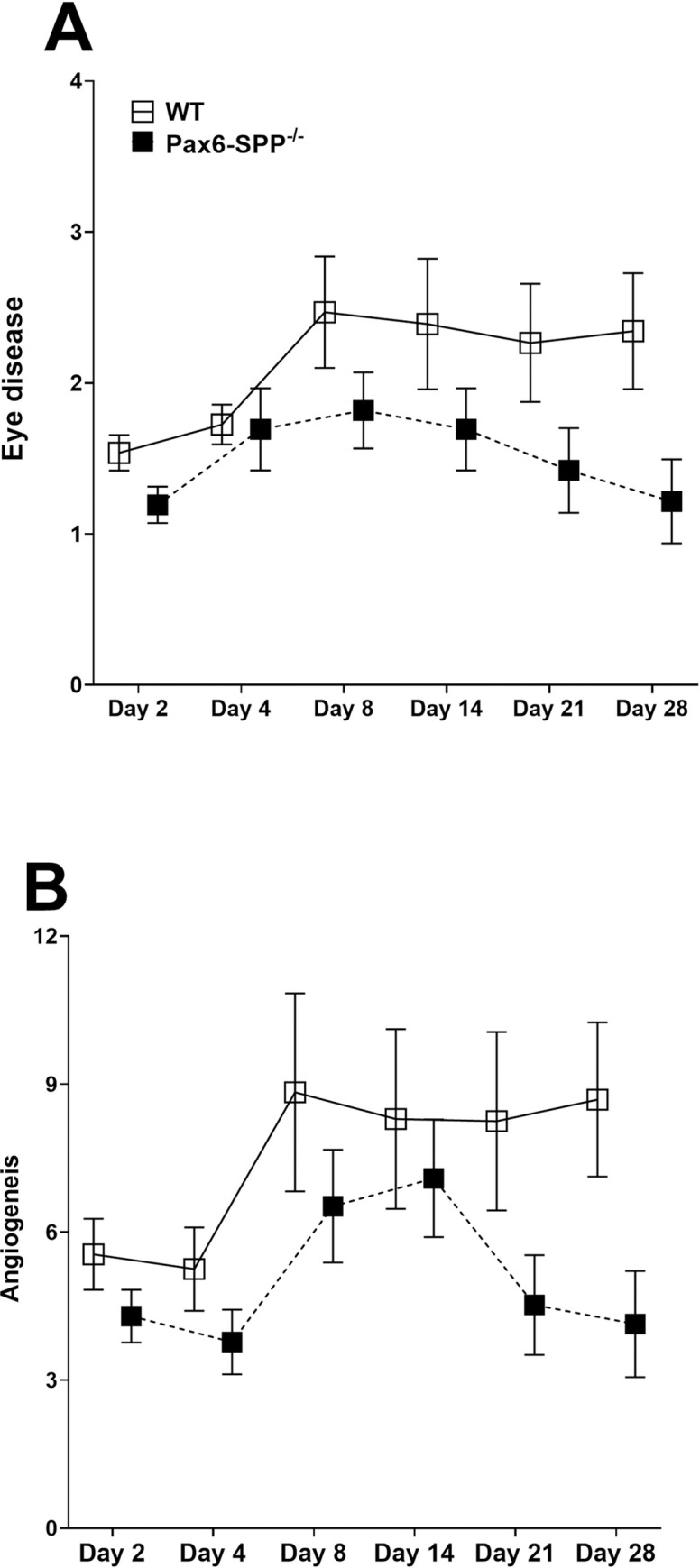
Eye disease and angiogenesis in infected mice. Pax6-SPP^-/-^ and WT control mice were ocularly infected with 2 X 10^5^ pfu/eye of HSV strain McKrae. Eye disease and angiogenesis were measured on days 2, 4, 8, 14, 21, and 28 under a slit-lamp as indicated in the Materials and Methods. Eye disease (panel A) and angiogenesis (panel B) are based on 20 eyes for WT mice (open boxes) and 22 eyes for Pax6-SPP^-/-^ mice (closed boxes).

To determine how the absence of SPP affects eye disease/corneal scarring and angiogenesis, infected Pax6-SPP^-/-^ and WT control mice described in Fig 5 above were scored for eye disease/corneal scarring and angiogenesis on days 2, 4, 8, 14, 21, and 28 PI as described in the Materials and Methods. No statistically significant differences in eye disease/corneal scarring were observed between Pax6-SPP^-/-^ and WT control mice on days 2, 4, 8 and 14 PI (Fig 6A, p>0.05, Fisher’s exact test), while eye disease/corneal scarring was significantly lower in Pax6-SPP^-/-^ mice than WT control mice on days 21 and 28 PI (Fig 6A, p<0.05, Fisher’s exact test). Similar to eye disease/corneal scarring (Fig 6A), angiogenesis was lower in Pax6-SPP^-/-^ mice than WT control mice on days 2, 4, 8, 14, 21, and 28 PI (Fig 6B). However, the differences were not statistically significant on days 2, 4, 8, and 14 PI (Fig 6B, p>0.05, Fisher’s exact test). However, on days 21 and 28 PI, Pax6-SPP^-/-^ mice had significantly lower levels of angiogenesis than did WT control mice (Fig 6B, p<0.05, Fisher’s exact test). These results suggest that absence of SPP in the eyes of infected mice does reduce the severity of eye disease/corneal scaring and angiogenesis. Similar to these results, we previously showed that inhibitors of SPP reduced eye disease in ocularly infected mice [20], while tamoxifen depletion of SPP in tamoxifen-inducible Cre mice [24] or deletion of SPP in peripheral sensory neurons did not affect eye disease in ocularly infected mice. Thus, deleting SPP reduces eye disease in infected mice suggesting that blocking the interaction of SPP with gK may provide therapeutic opportunities.

### Absence of SPP in the eye does not affect survival of infected mice

Survival over 4 weeks was monitored in four separate experiments using groups of 39 Pax6-SPP^-/-^ and 31 WT control mice that had been ocularly infected in both eyes with 2 X 10^5^ pfu/eye of McKrae virus. All 39 infected mice in the Pax6-SPP^-/-^ group survived ocular infection, while 28 of 31 mice in the WT control group survived ocular infection (p>0.05; ANOVA). These results suggest that the absence of SPP does not alter the survival of ocularly infected mice or WT control mice and it may have slightly, but not significantly, improved the survival of Pax6-SPP^-/-^ mice when compared with WT control mice.

### Absence of SPP in the eye does not alter latency or reactivation in TG of latently infected mice

**Fig 7:**
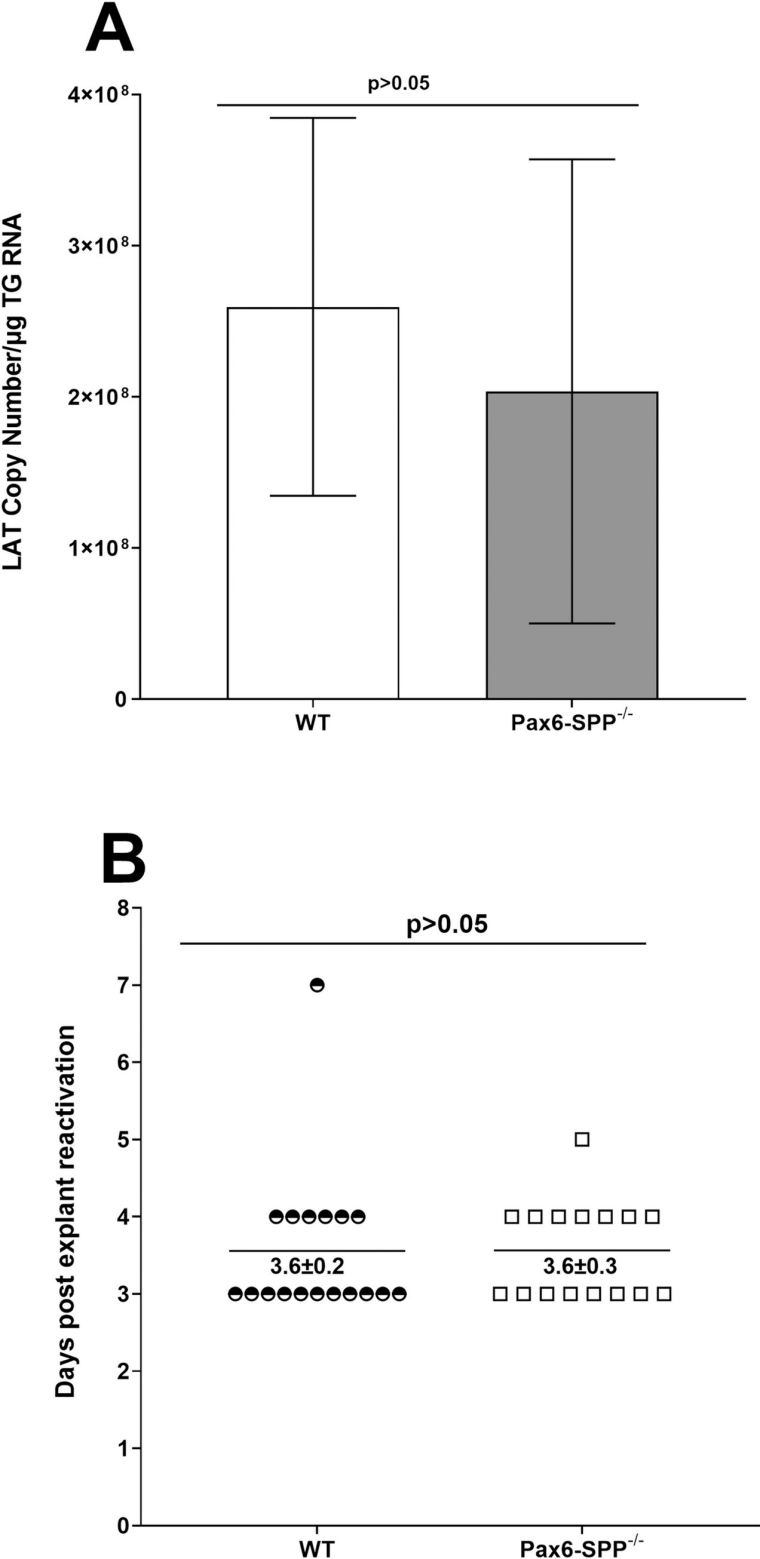
Effect of SPP deficiency on latency and reactivation in infected mice. A) LAT expression in TG of latently infected mice. Pax6-SPP^-/-^ (gray bars) and WT control (open bars) mice were ocularly infected with 2 X 10^5^ pfu/eye of HSV strain McKrae. TG were harvested at day 28 PI and LAT transcript levels were measured by qRT-PCR as described in Materials and Methods. Estimated relative LAT copy number was calculated using standard curves generated from pGEM-5317. GAPDH expression was used to normalize relative LAT RNA expression in the TG. Latency was determined using 12 TG for each group of mice: and (B) Explant reactivation in TG from latently infected mice. TG from latently infected mice were individually isolated on day 28 PI. Each individual TG was incubated in 1.5 ml of tissue culture media at 37°C. Media aliquots were removed from each culture daily and plated on indicator RS cells to assess the appearance of reactivated virus. Results are plotted as the number of TG that reactivated daily. Numbers indicate the average day that TG from each group first showed CPE ± SEM. Reactivation is based on 18 TG for WT mice and 16 TG for Pax6-SPP^-/-^ mice.

To determine whether the absence of SPP in the eyes of infected mice affects latency, Pax6-SPP^-/-^ and WT control mice were infected ocularly as above and TG from infected mice were isolated on day 28 PI. Total TG RNA was used to quantify LAT RNA copy number and cellular GAPDH RNA was used as an internal control. The amount of LAT RNA during latency in total TG extracts of Pax6-SPP^-/-^ mice was similar to that of WT control mice (Fig 7A; p>0.05, Fisher’s exact test). These results suggest that despite lower virus replication in the eyes of Pax6-SPP^-/-^ mice than in WT control mice, latency levels were similar in the two groups.

The qRT-PCR analyses described in Fig 7A suggested that the absence of SPP in eyes does not reduce LAT expression in TG of latently infected mice. Because LAT expression in TG was not affected during latency in Pax6-SPP^-/-^ mice, we asked whether reactivation of latent virus was similarly unaffected by the absence of SPP in the eye. To test this, Pax6-SPP^-/-^ and WT control mice were ocularly infected with 2 X 10^5^ pfu/eye of HSV strain McKrae. On day 28 PI, individual TG from infected mice were harvested and the kinetics of virus reactivation was measured in explanted TG. Average reactivation time for Pax6-SPP^-/-^ mice was 3.6 ± 0.3 days and WT control mice reactivated similarly at an average of 3.6 ± 0.2 days (Fig 7B, p = 0.9, Fisher’s exact test). Together, these results suggest that absence of SPP in the eye does not increase time to reactivation and also confirms our previous studies showing that lower virus load in the eyes of latently infected mice does not correlate with levels of latency or slower reactivation in TG of latently infected mice [35–37].

## Discussion

In susceptible mouse strains such as BALB/c as well as more refractory strains such as C57BL/6, there is a strong correlation between virus replication in the eye and increased eye disease following infection with virulent HSV strain McKrae [37–40]. Levels of virus replication in the eye and the severity of eye disease are also influenced by the degree of HSV neurovirulence. For example, avirulent HSV strains, such as KOS and RE, require corneal scarification for efficient ocular infection while virulent strains, such as McKrae, do not [10,41–45]. In addition to the degree of HSV virulence and mouse strains, corneal scarification also influences the level of virus replication in the eye and eye disease. Eye disease associated with HSV infection is facilitated by immune responses caused by the virus [3,5,46–49]. We have studied various HSV genes and found that immunization of mice with gK, but not with any other HSV gene, exacerbated eye disease and facial dermatitis independent of virus or mouse strain [7,8,10,18]. gK is an essential viral gene and HSV mutants that lack gK must be grown in gK-expressing cells [22,50–53]. A recombinant HSV that expressed two additional copies of the gK gene (rather than deleting the gK gene) exacerbated eye disease in two different strains of mice over that caused by the WT McKrae parental control [12].

Cell surface expression of gK is required to achieve elevated levels of CS in ocularly infected mice [17] and binding of gK to signal peptide peptidase (SPP), an endoplasmic reticulum protein, contributes to HSV infectivity *in vitro* and *in vivo* [19,20]. Thus, the interaction of gK with SPP facilitates its pathogenic functions *in vivo*. Since SPP is an essential gene, we previously used a tamoxifen-inducible Cre system to deplete SPP and showed that while SPP was not completely depleted, replication in the eye and eye disease both were decreased, and latency was reduced [24]. More recently we showed that deletion of SPP in peripheral sensory neurons reduced latency-reactivation in TG of infected Avil-SPP^-/-^ mice as compared with WT control mice but did not affect primary virus replication in the eye or eye disease in infected mice [28]. In contrast to the effect of tamoxifen-induced SPP depletion on virus replication in the eye and eye disease [24], we have shown that deletion of SPP in peripheral sensory neurons reduces latency-reactivation with no effect on virus replication in the eye or eye disease [28].

To determine the possible connection between primary virus replication in the eye and eye disease with the presence of SPP in the eye, in the present study, we specifically deleted SPP in the eye using the Pax6-Cre system. Pax6 is a highly conserved transcriptional factor that is essential for normal eye, central nervous system, and pancreatic development and maturation [54–57]. Previous studies showed that congenital aniridia (lack of an iris) in humans and murine small eye phenotypes, both arise from homologous defects in Pax6 [56,57]. In mice, the heterozygous Pax6 phenotype is associated with s*mall eye*, while *Pax6* homozygous mutant mice lack eyes and a nose and die after birth [58]. Pax6 is expressed in the lens, corneal epithelium, retinal neuroepithelium, and olfactory placodes/epithelium [59–62]. Pax6 is also expressed in various regions of the developing CNS [32]. Since, Pax6 is involved in eye development and is highly expressed in various eye tissues, using the Pax6-Cre system to delete SPP in the eye makes an excellent model to study eye infection in our model of ocular HSV infection. In this study we used a Pax6-Cre system to generate Pax6-SPP^-/-^ mice, which is an ideal model to directly evaluate the role of SPP during primary HSV infection. In contrast to the embryonic lethality of mice lacking SPP globally [23,24], deletion of SPP in Pax6-SPP^-/-^ mice had no obvious side effects. As expected, SPP expression was significantly lower in the eyes of Pax6-SPP^-/-^ mice than in WT control mice.

In contrast to our previous studies using chemical inhibitors to block SPP expression in WT mice, tamoxifen treatment to deplete SPP, or conditional knockout mice lacking SPP in their peripheral sensory neurons [19,24,28], the current study focused on blocking SPP expression in eye to specifically evaluate the role of SPP in virus replication in the eye of infected mice and its possible role in eye disease. We found that in the absence of SPP there was significantly less virus replication in the eye of infected Pax6-SPP^-/-^ mice than in WT control mice, which is consistent with our *in vivo* study of mice globally depleted of SPP after tamoxifen treatment [24] or treated with chemical SPP inhibitors [20]. We also showed that the absence of SPP in the eye is associated with significantly lower levels of gB, gK, and ICP0 transcripts in corneas of infected mice. We previously showed that global depletion of SPP was associated with significantly lower gB, gK, and ICP0 transcripts in infected mouse TG [24], while the absence of SPP specifically in the eye did not affect gB, gK, and ICP0 transcript levels in infected mouse TG. These differences were due to specific deletion of SPP in the eye without affecting its expression in the TG. Because Pax6 is expressed in the CNS [32], it is possible that deleting Pax6 could affect SPP expression in the CNS of infected mice. However, our results suggest that in contrast to depleting SPP in peripheral sensory neurons, which significantly reduced virus replication in the TG of Avil-SPP^-/-^ mice [28], deletion of SPP using Pax6 did not affect virus replication in infected mouse TG. Thus, the absence of SPP in the eye appears to reduce virus replication and viral transcripts in the eyes of Pax6-SPP^-/-^ mice without affecting virus replication in infected mouse TG. Similar to global SPP depletion by tamoxifen treatment [24] and the absence of SPP in peripheral sensory neurons of Avil-SPP^-/-^ mice [28], the absence of SPP in the eyes of infected Pax6-SPP^-/-^ mice did not affect their susceptibility to ocular infection with the virulent HSV strain McKrae.

The hallmark of HSV infection is corneal nerve degeneration and decreased corneal sensitivity in both human and animal studies [33,34,63,64]. In this study, the absence of SPP in the eye did not significantly affect corneal sensitivity to HSV infection, while WT control mice showed significantly reduced corneal sensitivity. Corneal sensitivity correlated with the presence of infectious virus and even after clearing virus from the eyes of infected mice, corneal sensitivity did not recover to that of infected Pax6-SPP^-/-^ mice. This decreased corneal sensitivity is consistent with previous work in which corneal sensation decreased between days 5–7 in infected mice, correlating with the presence of infectious virus in the eye [65]. Corneal nerve damage following HSV infection has been shown in humans and various mouse strains [64–69]. Thus, our results suggest that the absence of SPP prevents the interaction of gK with SPP in the eyes of infected Pax6-SPP^-/-^ mice, reduces virus replication, blocks nerve damage, and consequently block reducing corneal sensation to HSV-1 infection.

Pathology associated with HSV infection is a consequence of the host immune response mounted after virus infection [70]. The absence of SPP in infected Pax6-SPP^-/-^ mice was associated with less eye disease/corneal scarring and angiogenesis than in infected WT control mice. The reduced pathology in the eyes of infected Pax6-SPP^-/-^ mice directly correlated with virus titers in the eyes of infected mice. This finding is consistent with our previous study showing that virus load and duration of primary virus replication in the eye are directly related to the severity of eye disease in ocularly infected mice [7,8,10]. In contrast to this study, depleting SPP by tamoxifen treatment did not reduce eye disease in ocularly infected mice [24]. The discrepancy between our current study and our tamoxifen depletion study is probably due to incomplete SPP depletion by tamoxifen treatment because even after seven tamoxifen treatments, we only depleted 93% of SPP in treated mice [24]. This lack of complete SPP depletion is why we conditionally deleted SPP in the eye using Pax6-SPP^-/-^ mice and in peripheral sensory neurons using Avil-SPP^-/-^ mice [28]. However, the absence of SPP in peripheral nerves of Avil-SPP^-/-^ mice did not affect eye disease [28]. These results suggest that the effect of SPP absence on virus replication in the eye and TG depends on the organ SPP was deleted from.

In this study we found similar levels of LAT expression in TG of Pax6-SPP^-/-^ mice and in WT control mice. This is in contrast to when we depleted SPP using tamoxifen [24] or used Avil-SPP^-/-^ mice lacking SPP in their peripheral sensory neurons [28]. Differences between our current and previous studies are associated with site of SPP depletion. In the current study we specifically deleted SPP in eye tissue without affecting SPP expression in the TG. Further, the absence of SPP expression in the eyes of Pax6-SPP^-/-^ mice did not delay the time of reactivation in infected Pax6-SPP^-/-^ mice. These results contrast with our recent study in Avil-SPP^-/-^ mice lacking SPP in their peripheral sensory neurons, which also had lower reactivation than WT control mice [28]. Taken together, this study and our previous studies [24,28] suggest that effect of SPP on virus replication, eye disease, latency, and reactivation depends on what tissue SPP has been deleted from.

In summary, we have provided the first evidence that the absence of SPP specifically in the eyes of infected Pax6-SPP^-/-^ mice reduces virus replication in eye, reduces corneal sensitivity, reduces eye disease/corneal scarring and angiogenesis compared with WT control mice. However, the absence of SPP in the eye did not alter levels of latency-reactivation in TG of infected Pax6-SPP^-/-^ mice compared with WT control mice. Based on our current and previous studies, we conclude that the interaction of gK with SPP regulates both primary and latent infection in a tissue-dependent manner. Consequently, blocking this interaction may have both prophylactic and therapeutic potentials.

## Material and methods

### Ethics statement

All animal procedures were performed in strict accordance with the Association for Research in Vision and Ophthalmology Statement for the Use of Animals in Ophthalmic and Vision Research and the NIH guide for Care and Use of Laboratory Animals (ISBN 0-309-05377-3). The animal research protocol was approved by the Institutional Animal Care and Use Committee of Cedars-Sinai Medical Center (Protocol # 9129).

### Cells and virus

The rabbit skin (RS) cell line was cultured in minimal essential medium plus 5% fetal bovine serum and maintained as described previously. Triple-plaque-purified HSV strain McKrae was grown in RS cell monolayers as described previously [8,71]. HSV strain McKrae is a virulent strain of virus that infects mice efficiently without corneal scarification.

### Generation of Pax6-SPP^-/-^ mice

SPP^flox/flox^ mice in C57BL/6 background were generated and maintained in-house as we previously described [24,28]. Pax6-SPP^-/-^ conditional knockout mice were generated by crossing SPP^flox/flox^ mice with Pax6-Cre mice that have intact Pax6 expression (#024688; Jackson Laboratory). The Cre that we used in Pax6-Cre mice is under Pax6 promoter enhancer and Pax6-SPP^-/-^ conditional knockout mice have intact Pax6 expression [29,30]. Female Cre mice are leaky, thus only male Pax6-SPP^-/-^ mice were used to breed with female SPP^flox/flox^ to generate pups. Pax6-Cre positive mice were identified by PCR genotyping and used in studies described below. WT C57BL/6 mice were purchased from The Jackson Laboratory (Bar Harbor, ME) and bred in-house at Cedars-Sinai Medical Center for use as controls.

### Primers for Pax6-SPP^-/-^ mice genotyping

The following primers were used to screen Pax6-SPP^-/-^mice: SPP flox forward primer: TGCCTCCCGTTTAAGAGACC; SPP flox reverse primer, GACTCATTCTC CCCGCTCTG. The wild-type allele product length is 605 bp with a floxed allele product length of 688 bp. The following primers were used for Pax6-Cre: CACCAGAGACGGAAATCCATC (forward) and GGCCAGCTAAACATGCTTCA (reverse) with a PCR product length of 400 bp. The following primers were used for the SPP deletion: CGCTTCGGAGCATTGTGA (forward) and CCATGAGGAGCCAAGCC (reverse) with a PCR product length of 270 bp.

### Ocular infection

WT control and Pax6-SPP^-/-^ mice (7 to 8 weeks old of both sexes) were ocularly infected in both eyes with 2 X 10^5^ pfu/eye of HSV strain McKrae in MEM medium as an eye drop without corneal scarification as we described previously [8].

### RNA extraction and cDNA preparation

Corneas or TG from infected mice were harvested at indicated times. Collected tissues were immersed in TRIzol reagent and stored at −80°C until processing. Corneas and TG from each animal were processed for RNA extraction as described previously [12]. Isolated total RNA was reverse transcribed using high capacity cDNA reverse transcription kit (Applied Biosystems, Foster City, CA) according to the manufacturer’s recommendations.

### TaqMan quantitative RT-PCR

Expression levels of the gB, gK, ICP0, CD4, CD8α, and IFNγ transcripts were evaluated using custom-made TaqMan primer sets as follows: for gB, 5′-AACGCGA CGCACATCAAG-3′ (forward), 5′-CTGGTACGCGATCAGAAAGC-3′ (reverse), and probe 5’ FAM-CAG CCGCAGTACTACC-3′ (where FAM is 6-carboxyfluorescein); for gK, For gK, 5’-GGCCACCTACC TCTT GAACTAC-3’ (forward), 5′-CAGGCGGGTAATTTTCGTGTAG-3′ (reverse), and probe 5′-FAM-CAGGCC GCATCGTATC-3; and for ICP0, 5′-CGGACACGGAACTGTTCGA-3′ (forward), 5′-CGCCCCCGCAACTG-3′ (reverse), and probe 5′-FAM-CCCCATCCACGCCCTG-3′. GAPDH primers from Applied Biosystems (assay identifier [ID], m999999.15_G1) were used as an internal control. Other TaqMan primers from Applied Biosystems used in this study included CD8α (Mm01182107_g1), IFNγ (Mm01168134_m1), and CD4 (Mm00442754_m1).

In each experiment, the estimated relative copy number of gB, gK, and ICP0 genes were calculated using standard curves generated from plasmids containing the gene of interest: pGem-gK (for gK) [7], pAc-gB1 (for gB) [72] and pcDNA-ICP0 (for ICP0) [73]. Briefly, each plasmid DNA template was serially diluted 10-fold so that 1 μl contained from 10^3^ to 10^8^ copies of the desired gene and then was subjected to TaqMan PCR with the same set of primers as the test samples. The copy number for each reaction product was determined by comparing the normalized threshold cycle (*C*_*T*_) of each sample to the threshold cycle of the standard curve. To analyze fold change of expression, the 2^−ΔΔ*CT*^ method was used to calculate gene expression fold change compared to expression in uninfected controls in each group.

### IHC staining

Eyes from Pax6-SPP^-/-^ and WT control mice at around two months of age were harvested, frozen in OCT compound, and stored at -80°C until further processing. Frozen tissue was sectioned using a Cryostar NX70 (Thermo Scientific, Rockford, IL). Tissue sections were fixed with 4% paraformaldehyde, washed with 1× phosphate-buffered saline (PBS), and then permeabilized with 0.3% Triton X-100 in PBS. The slides were blocked using 1× sea blocker (Thermo Scientific) plus 5% horse serum for 1 hr at 25°C, then incubated with anti-SPP antibody (Bethyl Laboratory, A304-404A, Waltham, MA) in blocking buffer at 4°C overnight. Slides were washed three times with 1XPBS and incubated with Biotin-conjugated secondary antibody for 2 hr at 25°C. Slides were then washed four times with 1xPBS and developed using a Vector VIP substrate kit according to company instruction (Vector Laboratories, Burlingame, CA. Cat# SK-4600). Vector Methyl green was used for counter staining. Slides were mounted with Permount mounting medium (Thermo Scientific, SP15-100) and image acquisition and data analysis were performed using a Leica DM4000 microscope (Leica Microsystems, Buffalo Grove, IL). SPP staining was quantified using Image J software.

### Titration of HSV-1 in eye

Tear films were collected from both eyes of infected mice on days 1 to 5 after infection using a cotton applicator. Each swab was placed in 1 ml of tissue culture medium and stored at –80°C until processing. The amount of virus in the medium was determined by a standard plaque assay using RS cells as we previous reported [10].

### HSV-induced eye disease and angiogenesis

The severity of corneal scarring/corneal disease lesions in mouse corneas was examined by slit lamp biomicroscopy on days 2, 4, 8, 14, 21 and 28 PI. The examination was conducted by an investigator blinded to the treatment regimen of the mice. The mice were randomly screened per group without anesthesia for the haziness. Scoring scale was: 0, normal cornea; 1, mild haze; 2, moderate opacity; 3, severe corneal opacity but iris visible; 4, opaque and corneal ulcer; 5, corneal rupture and necrotizing keratitis, as we previously described previously [10,74]. The severity of angiogenesis on day 28 PI was recorded using on a 4 pt scale in which a grade of 4 for a given quadrant of the circle represents a centripetal growth of 1.5 mm toward the corneal center. Scores of the four quadrants of the eye were summed to derive the neovessel index (range, 0–16) for each eye at a given time point. The data was then plotted into prism to generate a graph.

### HSV-induced corneal sensitivity

Corneal sensitivity post infection was measured by a Cochet Bonnet aesthesiometer at indicated days post infection as described previously [75]. Briefly, starting from the longest filament length of Cochet Bonnet aesthesiometer (6 inches in our case), the center area of cornea was touched, and the length of the filament was recorded when the touch caused a blink within three tries, otherwise the filament of the aesthesiometer was shorten by 0.5 inch and the experiment was repeated. The recorded filament length was an indication of the sensitivity of cornea and thus, longer filament lengths being associated with higher corneal sensitivity.

### Determining the level of latency in TG of latently infected mice

TG from individual mice were collected on day 28 PI, immersed in RNA stabilization reagent (RNA Later, Thermo Fisher Scientific, Waltham, MA, USA), and stored at −80°C until processing. Total RNA was extracted as we described previously [76,77]. LAT RNA levels from latent TG were determined using custom-made LAT primers and probe as follows: forward primer, 5′-GGGTGGGCTCGTGTTACAG-3′; reverse primer, 5′-GGACGGGTAAGTAACAGAGTCTCTA-3′; and probe, 5′-FAM-ACACCAGCCCGTTC TTT-3′ (amplicon length = 81 bp). GAPDH was used as a loading control. LAT RNA copy number was calculated using a standard curve generated using pGEM5317-LAT as we described previously [78].

### *In vitro* explant reactivation assay

TG from infected mice were harvested on day 28 PI and cultured in 1.5 ml tissue culture medium. The reactivation assay was performed as we described previously [12,79]. Briefly, a 100-μl aliquot was taken from each culture daily and used to infect RS cell monolayers. The RS cells were monitored daily for the appearance of cytopathic effect (CPE) for 5 days to determine the time at which reactivated virus first appeared from each TG. As the media from the explanted TG cultures were plated daily, the time at which reactivated virus first appeared in the explanted TG cultures could be determined.

### Statistical analysis

Fisher’s exact test and ANOVA test were performed using the computer program Instat (GraphPad, San Diego, CA). Results were considered statistically significant at a *P* value of <0.05. All experiments were repeated at least two times to ensure accuracy.
